# Diabetes Management Through Remote Patient Monitoring: A Mixed-Methods Evaluation of Program Enrollment and Attrition

**DOI:** 10.3390/healthcare13070698

**Published:** 2025-03-22

**Authors:** Dejun Su, Tzeyu L. Michaud, Jessica Ern, Jian Li, Liwei Chen, Yan Li, Lu Shi, Donglan Zhang, Jennifer Andersen, José A. Pagán

**Affiliations:** 1 Department of Health Promotion, College of Public Health, University of Nebraska Medical Center, Omaha, NE 68198, USA; tzeyu.michaud@unmc.edu; 2 Center for Reducing Health Disparities, College of Public Health, University of Nebraska Medical Center, Omaha, NE 68198, USA; jessicaern44@gmail.com; 3 Department of Environmental Health Sciences, Fielding School of Public Health, University of California Los Angeles, Los Angeles, CA 90095, USA; jianli2019@g.ucla.edu; 4 Department of Epidemiology, Fielding School of Public Health, University of California Los Angeles, Los Angeles, CA 90095, USA; cliwei86@g.ucla.edu; 5 School of Medicine, Shanghai Jiao Tong University, Shanghai 200025, China; yanli2022@sjtu.edu.cn; 6 College of Health Professions, Pace University, New York, NY 10038, USA; lshi@pace.edu; 7 Division of Health Services Research, Department of Foundations of Medicine, New York University Grossman Long Island School of Medicine, Mineola, NY 11501, USA; donglan.zhang@nyulangone.org; 8 Department of Internal Medicine, Division of Community Health & Research, University of Arkansas for Medical Sciences Northwest, Springdale, AR 72762, USA; jaandersen@uams.edu; 9 Department of Public Health Policy and Management, School of Global Public Health, New York University, New York, NY 10003, USA; jose.pagan@nyu.edu

**Keywords:** remote patient monitoring, diabetes management, program reach, program enrollment, program attrition, mixed methods

## Abstract

**Background:** Despite the growing use of remote patient monitoring (RPM) in diabetes management, few studies have assessed program enrollment and attrition. This study adopted a mixed-methods approach to examining factors linked to program enrollment and attrition amongst a large sample of patients who went through RPM in diabetes management. **Methods:** Based on quantitative data from the Remote Interventions Improving Specialty Complex Care program conducted in Nebraska from 2014 to 2018, chi-squared or *t* tests were used to compare three groups of patients with diabetes who had been contacted for program participation: those who completed the intervention, withdrew from the intervention, or declined to participate. Logistic regression was used to identify factors associated with program dropout. Inductive thematic analysis was conducted to assess patient feedback based on semi-structured interviews with patients from the three groups. **Results:** Out of the 1993 patients with diabetes invited for participation, 13% (n = 256) declined to participate, 16% (n = 317) withdrew before completion, and 71% (n = 1420) completed the intervention. Being younger or having poorer health (as indicated by higher blood glucose or blood pressure) at the baseline was associated with higher odds of program withdrawal. The top reason patients cited for declining participation or withdrawal from RPM was not having enough time to complete the intervention. Patients who declined to participate mentioned that an offer of incentives or more information at the beginning of the intervention may increase their motivation for participation. **Conclusions:** Being younger or having poorer health at the baseline was associated with higher odds of withdrawing from the RPM program. Future RPM programs can increase program retention by becoming more responsive to the health needs of vulnerable patients who struggle with managing their diabetes or related comorbidities at the baseline.

## 1. Introduction

Remote patient monitoring (RPM), defined as “the recording and transmission of patient biometrics, vital signs and/or disease-related data to a healthcare provider using information and communications technology [1]”, has been increasingly adopted in diabetes management [2]. This trend has recently been boosted by the COVID-19 pandemic, giving rise to increasing demand for RPM as a result of medical urgency, technology advances, and expanded reimbursements and policy support for RPM [3]. An examination of Medicare claims data revealed that the utilization of general RPM among Medicare beneficiaries increased by 555% from February 2020 to September 2021 [4]. About 43% of adults in the United States reported the use of telehealth including RPM during COVID-19 [5].

The growing use of RPM, however, does not necessarily mean that all patients with diabetes have the motivation to participate in RPM and potentially benefit from it. Although some patients might appreciate the convenience of digital health technologies for disease monitoring (e.g., saving travel time and costs), others might perceive using these technologies as a burden and would avoid them because of a lack of understanding of the technology or a preference for physical over virtual encounters with healthcare providers [6]. Understanding how these factors influence RPM participation among patients with diabetes can be useful to develop targeted interventions to engage patients more effectively while increasing program reach and retention.

Patient retention in RPM requires consistent and continuous effort throughout the intervention period. The long duration of RPM in diabetes management has made effective retention of program participants a significant challenge [7,8]. A systematic review of clinical interventions based on remote monitoring and coaching of diabetes management revealed that attrition rates ranged from 9% to 21% [9]. The overall attrition rates in app-based, remote digital health interventions targeting diverse chronic conditions were even higher [8,9,10].

Despite the documented effectiveness of RPM in diabetes management [11,12], few studies have examined the willingness of patients to participate in or complete RPM. One recent study investigated the most common reasons for continuous glucose monitoring attrition in 2663 youth with type 1 diabetes [13]. The findings suggested that those who discontinued the monitoring tended to be older, have had longer disease duration or higher A1C, and self-identify as non-Hispanic Black. The most common reasons cited for attrition were problems with device adhesion, disliking a device on the body, insurance problems, pain with device use, and system mistrust due to inaccurate readings. While these findings are important for understanding factors linked to attrition in glucose monitoring for youths with type 1 diabetes, it remains unclear whether these findings can be extended to older patients or patients with type 2 diabetes.

Part of the challenges in identifying factors associated with program enrollment or attrition in RPM for diabetes management concerns the lack of comprehensive data. Collecting quantitative or qualitative data from patients who declined to participate in RPM or who decided to quit before program completion is understandably more difficult, which poses a significant challenge in assessing the reach and impact of RPM in diabetes management [14]. This study used mixed methods to assess program enrollment and attrition in a large RPM program serving patients with type 2 diabetes (T2D).

## 2. Materials and Methods

### 2.1. Study Setting and Sample

Data used in this study came from the Remote Interventions Improving Specialty Complex Care (RIISCC) program implemented at Nebraska Medicine, the top rated hospital in the State of Nebraska, from 2014 to 2018. While the quantitative data from this program have been previously analyzed for research purpose, the qualitative data from this program have never been used for research. Funded by a Health Care Innovation Award from the Centers for Medicare & Medicaid Services, RIISCC aimed to improve clinical outcomes and reduce healthcare costs for patients with T2D discharged from Nebraska Medicine—including patients with comorbidities such as congestive heart failure, hypertension, and acute myocardial infarction. Patients with T2D who had a recent hospital admission for any reason were recruited to the program no later than one month after hospital discharge. Specifically, inclusion criteria included: (1) diagnosis of T2D; (2) 19 years of age or older (legal age in Nebraska); (3) able to use glucometer (or provided with and taught to use a meter compatible with the equipment provided) and take insulin and/or other prescribed medications; (4) not pregnant; (5) not having a history of substance use disorder; (6) English-speaking and able to read English (by herself/himself or with help from others); (7) discharge planned to home; and (8) able to express a basic understanding of and successfully use RPM equipment. During the program period, the clinical team in RIISCC contacted a total of 1993 eligible patients and shared with them information about the program. RIISCC was exempt from IRB approval since it was set up as a hospital-based quality improvement program. To protect the privacy of participating patients, all program data had been anonymized before they were used for this study.

**Figure 1:**
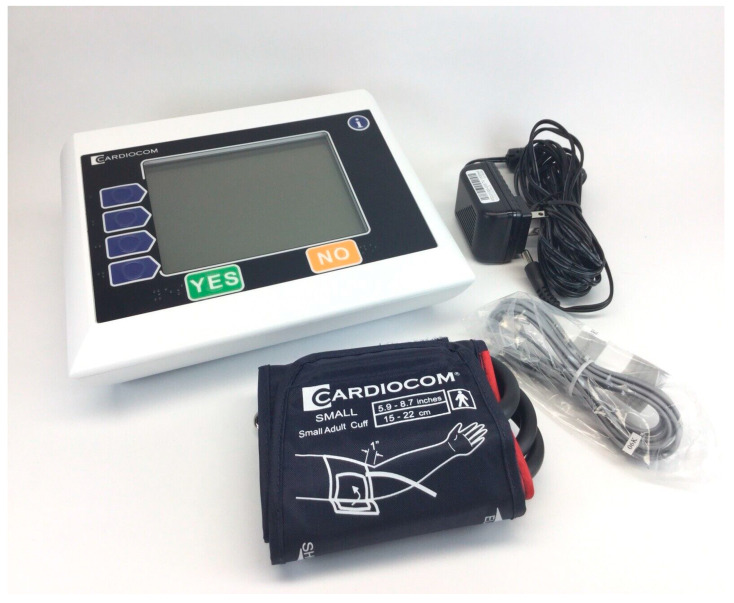
Cardiocom equipment used in the RPM program.

After a patient was enrolled, a medical assistant would visit the patient at their home to set up the RPM equipment (Cardiocom, Medtronic Inc., Minneapolis, MN, USA) including a cellular base unit, blood pressure cuff, blood glucose meter, weight scale, and necessary cords, as partially indicated by Figure 1. During the home visit, the medical assistant would teach the patient how to use the equipment and upload data for monitoring. This was then followed by a 3-month intervention involving daily remote monitoring of blood pressure, weight, and glucose, and a weekly phone call from an assigned nurse. Participating patients were expected to take the biometric measurement on a daily basis during the intervention and had access to their nurse coaches via email or phone as needed. Ten nurse coaches (all female) provided services including medication adherence assessments, nutritional counseling, and diabetes self-management coaching and education. The nurse coaches would call patients whenever the uploaded data triggered a medical alert or emergency. Primary care providers were provided with patient data uploaded throughout the 3-month intervention via electronic health records. At the end of the intervention, patients were seen at a local community health center where they returned the RPM equipment and received diabetic retinopathy screening, hemoglobin A1C (HbA1c) testing, a virtual nutritional counseling session, and a feet exam from a certified diabetes educator (assisted by an on-site medical assistant) [12]. A simple courtesy telephone call was made on a monthly basis for an additional 9 months after participants had concluded the program.

Financial incentives were used in RIISCC to promote program retention. Enrolled patients who successfully completed the program would receive gift cards with a total value of USD 50, distributed as: (1) USD 10 gift card 7 days after first upload, (2) USD 10 gift card for continuous monitoring 30 days after first upload, (3) USD 10 gift card for continuous monitoring 60 days after first upload, and (4) USD 10 gift card for completing the 90-day intervention and having their HbA1c test done at their assigned community health center or clinic, as well as a USD 10 gift card for returning their RPM equipment.

### 2.2. Measures

#### 2.2.1. Quantitative Data

The quantitative data in RIISCC contained information on three health outcomes including HbA1c, high blood pressure (>140/90 mmHg or not), and bodyweight in pounds. Data for these measures were collected at both baseline and the end of the intervention for patients who completed the intervention, allowing for an assessment of changes in these health outcomes.

The Patient Activation Measure-13 (PAM-13) [15] was used to denote the degree of patient activation at both baseline and the end of three months of RPM. PAM-13 is a uni-dimensional, interval level, Guttman-like scale consisting of 13-item questions used to measure patient knowledge, skills, and confidence for self-management of chronic conditions. Subsequent work on this scale further differentiated patients into four levels of activation based on the scoring of their responses: Level 1—the patient does not yet believe they are active or have an important role in managing their health (<47.0); Level 2—the patient lacks confidence and knowledge to take action to manage their health (47.1–55.1); Level 3—the patient is beginning to take action to manage their health (55.2–67.0); and Level 4—the patient is maintaining actions of managing their health over time (≥67.1) [16].

Demographic data included age at baseline in years, sex (male, female), and race (non-Hispanic White vs. racial and ethnic minorities). RIISCC also tracked the number of uploads of biometric data by each enrolled patient, an indicator of patient engagement in the program [17].

#### 2.2.2. Qualitative Data

**Figure 2:**
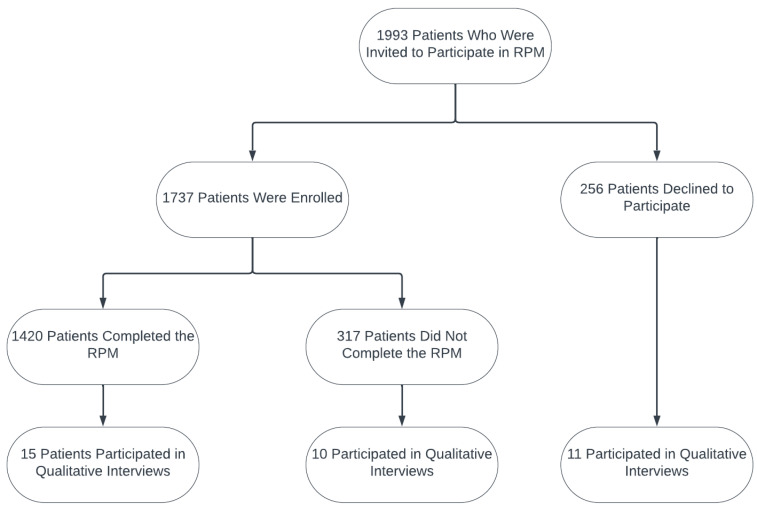
Data components and related sample sizes in the study.

In an effort to qualitatively identify factors affecting program reach, retention, and effectiveness, the RIISCC team trained and supported a Clinical Research Outreach Coordinator (CROC) to interview a convenience sample of 36 patients with T2D over the phone in January 2017, including 15 patients who completed the 3-month RPM, 10 patients who were enrolled but chose to withdraw from the program with no completion, and 11 eligible patients who declined to participate in the program. Figure 2 illustrates the data components, sample sizes, and the linkage between the quantitative and qualitative data used in the study.

These semi-structured interviews were conducted in English, with each interview typically lasting between 10 and 15 min. Before starting each telephone interview, the CROC explained to the patient the purpose of the interview, its expected length, the needed recording of the interview, and a modest incentive (USD 20) offered to compensate the patient for their time. After receiving a clear approval from the patient, the CROC then proceeded with the interview and its recording.

The evaluation team in RIISCC initially drafted the questions for interviews in each of the three groups of patients before finalizing them with input from the rest of the RIISCC team. Table 1 listed the questions used in the interviews. Although the interviews with patients who had completed the program focused on patient feedback on their experience in the program, the core of the interviews with the other two groups of patients revolved around why they chose to withdraw from the program before completion or to decline to participate in the program, and the potential strategies to retain or motivate their participation.

### 2.3. Data Analysis

The quantitative analysis in this study started with a comparative analysis of explanatory variables across the three groups of patients including those who completed the 3-month RPM intervention (the Completed group), those who participated in the intervention but chose to quit the intervention before its completion (the Withdraw group), and those who declined to participate in the intervention (the Declined group). Of note, no data other than age, sex, race, and health insurance status was collected for the Declined group.

Chi-squared tests, independent sample *t*-tests or one-way ANOVA (only used for the age comparison across three groups) were used to denote whether the differences across groups were statistically significant. Among the Completed group, paired *t*-tests or chi-square tests were estimated to assess if the changes in health outcomes between the baseline and the end of the intervention were statistically significant. This was followed by a logistic regression to identify factors associated with sample attrition among all enrolled patients (the Withdraw group vs. the Completed group). All analyses were conducted using Stata version 14. Two-tailed *p* values of less than 0.05 were considered statistically significant.

For the qualitative data analysis, all interviews with patients were recorded, fully transcribed verbatim, and compared to the recording for accuracy. The study team evaluated the data using inductive thematic analysis to develop a codebook [18]. The study team assessed saturation of the data by examining the scope of the topics covered and the extent to which additional interviews might yield new insights before deciding to stop participant recruitment. The qualitative data from each of the three patient groups (Completed, Withdraw, or Declined) were coded separately. Initial codes were grouped into two overarching themes: positive and negative perception of the RPM program. Using the codebook, a paired analysis of the data was conducted by two researchers. To evaluate the consistency between the two researchers in their coding, the Cohen’s kappa statistic was estimated, which yielded an interrater reliability of 0.937, suggesting strong consistency between the two researchers [19].

## 3. Results

### 3.1. A Comparison Across the Three Patient Groups

Among the 1993 patients with T2D who were invited to participate in the RIISCC program, 1737 enrolled (87%) and 256 (13%) declined to participate. Among the enrolled participants, 1420 (82%) completed the 3-month intervention and 317 (18%) did not complete the program. A comparison across the three groups, as summarized in Table 2, revealed several significant differences in demographics. The average age of patients in the completed group was notably older than those in the Withdrawal group (59.5 years versus 54.5 years). The proportion of females in the Declined group (68%) was substantially higher than those in the other two groups. In terms of racial composition, the proportion of racial minorities was 45% in the Declined group, as compared to 40% in the Withdrawal group and 32% in the Completed group.

Available data also revealed significant differences between the Completed and Withdrawal groups in terms of baseline health status. Overall, patients in the Withdrawal group were less healthy than those in the completed group, as indicated by higher average HbA1c, higher percentage of patients with HbA1c greater than 9% at the baseline, as well as higher percentage of patients with hypertension. The two groups, however, did not have significant differences in patient activation in diabetes management at the baseline.

### 3.2. Feedback from Patients Who Declined to Participate in the Program

Among the 256 patients who declined to participate in the RIISCC program, 11 agreed to participate in the interview. In terms of demographic composition, two of the interviewees were African American and nine were non-Hispanic White; five were female and six were male.

Six patients stated they were approached regarding the RIISCC program from a phone invitation, and three were approached during a hospital stay. Ten patients stated they had sufficient information they needed to make an informed decision regarding participation, but external factors, such as being too overwhelmed at the time, or participation in other activities (e.g., work, childcare, other health programs), had thwarted their participation, as exemplified by the following sample statements.“It’s timing. It was just a lot going on with kids and school and them running here and there and everything else so.”“My life is crazy, I mean just nuts. We got a kid in Minnesota in college. We make it up there at least once a month, you know what I’m saying. And I work seven days a week.”“I declined to participate because I was already doing something similar through the Veteran Health Administration. I even have a scale too.”

Patients who declined to participate mentioned several additional factors that may increase their chance of participation, including the use of incentives, more information provided at the beginning, or at a time after the hospital discharge, and less time-consuming overall.“I probably would have participated if you had waited until when I was at home… I was pretty overwhelmed with everything.”“Well one thing to get people faster, it would be to throw a little money in there. That would be my advice because that would help a lot of people say yes if they had a little bit of incentive to do so.”“Just to kind of explain it at the beginning and let people know what it is and when you’re available to do it and you have the time… what dates are coming.”

### 3.3. Factors Associated with Program Attrition

As Table 3 shows, several factors were significantly associated with the odds of dropping out of the RIISCC program before completion. For each additional year in age, the odds of withdrawing from the program decreased after adjusting for the effect of other covariates in the model (adjusted odds ratio [AOR] = 0.97, 95% confidence interval [CI]: 0.96–0.98). Poor health at baseline was associated with higher odds of program attrition. Each one unit of increase in HbA1c at the baseline was associated with higher odds of withdrawal from the program (AOR = 1.07, 95% CI: 1.01–1.14). Relative to patients who did not have hypertension at the baseline, the odds for patients with hypertension to withdraw from the program were higher (AOR = 1.39, 95% CI: 1.05–1.84).

Among the patients who withdrew from the program, 10 of them (7 males and 3 females; 5 African American patients and 5 non-Hispanic White patients) were able to participate in the telephone interview to share their perspectives. The most common reasons for withdrawing from the program involved personal lifestyle issues, such as moving and hectic schedules, as exemplified by the following quotes.“I liked the whole program. I just wasn’t devoted to doing it. It was just, I was just a busy person. I was going through some things and I just couldn’t keep committed to doing it on a regular basis.”“It was just the fact that it was kind of inconvenient for me to have to do every morning because I had a lot of company so I didn’t want to continue it.”“It was inconvenient to me. I was going to be traveling and I couldn’t …I didn’t want to have to carry the equipment around and then I kind of like to keep my weight to myself and I didn’t want everybody to hear because you know the monitor would always speak out loud to what it was, so I didn’t want to have to worry about that.”

Patients in this group expressed concerns about technological issues they had encountered when using the RPM equipment at home including its functioning and accuracy.“When I get read to do the glucose, sometimes it wouldn’t go into the machine.”“I’d say on the scale, the scale was…you know it’s hard to recall. I’m going to say five to ten pounds off. And the blood pressure was way off. I couldn’t even tell you the blood pressure but it wasn’t accurate at all.”

Several patients recommended specific changes to the program they would like to see such as program duration, adjustable voice control on the monitor, and interactions with nurse practitioners, as illustrated by the following quotes.“I would have liked to be monitored longer than the 90 days thing or whatever it was.”“If it was something that could be changed, the one thing would be the voice on the thing, I could do that so that I could turn it down or something you know. Have an adjustment on the monitor or something so you could turn the sound up and down or maybe the way that it kept repeating the instructions and getting started…that was a slow process to start with.”“I think with different equipment and they could answer some of my questions or let me interface with maybe, let’s say once a month with a nurse practitioner, who may have more experience with diabetics, I think that would be great.”

Overall, patients who had withdrawn from the program were positive about the benefits of the program. Seven of the 10 patients from this group stated they would participate again when the program was offered in the future, with one patient being unsure of future participation and two patients stating they would not participate in the future.

### 3.4. Program Outcomes Based on Data from the Patients Who Completed the Intervention

As Table 4 shows, patients in the Completed group demonstrated several positive changes in selected outcomes between the baseline and the end of the intervention. This can be illustrated by the substantial, significant reduction in average HbA1c. There was also a significant, though slight, reduction in bodyweight. The intervention witnessed a significant increase in patient activation in diabetes management. However, the proportion of patients with hypertension experienced a significant increase.

Qualitative feedback based on interviews with 15 patients who completed the intervention provided insights into patient perception of the intervention. Of these 15 participants, 4 (26.7%) were African Americans and 9 were female (60%). All 15 participants stated health improvement was the motive to participate and to continue to participate. Some examples of this include:“I think this is, especially for people who may not be as in tune to the habit of checking their weight, checking their blood sugar, and checking their blood pressure on a regular basis, I think that this would be a good way for people doing that.”“…it helps me be more aware of my health.”“…just monitoring and it kept track of everything. I liked that.”

Among the participants who completed the intervention program, 14 participants had a positive experience and 1 individual had a neutral experience. In this group, the most common positive associations with the program included simple and intuitive technology and increased communications. Examples of improved communication include:“I really liked it! What I really liked was that they get it all done at one time. Get up…you know, it just syncs right into a habit. Get up, weigh yourself, take your blood pressure, take your blood sugar. I really liked that.”“I had a couple of not quite official low blood sugars but low enough that they caused symptoms of low blood sugar. And so my doctors were able to see that and chat with me, as were the people involved in the research monitoring the numbers and so that was really good to be able to have that immediate feedback.”

Sample quotes for technology appreciation include:“I liked it fine. It seemed to work pretty well. I didn’t have any problems.”“I really enjoyed that cuffing scale… Everything worked like clockwork.”“It was super easy. I just kept it in my bathroom and used it every day. You know, just stepping on the scale and then just putting the thing on my arm.”

Although the response was overwhelmingly positive among the participants who completed the program, there were some negative aspects participants mentioned. The majority of these issues arose with technology difficulties or aspects of the technology, as indicated by the following quotes.“…sometimes it’d be like some glitches in the equipment. Like a couple times, it didn’t record my blood sugar readings and then like another couple times, it didn’t record my weight.”“I didn’t ask but it’s no big deal, but I couldn’t figure out how to turn the volume down on the woman’s voice.”“Obviously, after a period of amount of time, it gets repetitious but like I said, it was very easy and self-explanatory, more than anything.”

Other negative perceptions pertained to availability of study staff and lack of general results being available. Examples of this are as follows:“It was just harder to make contact at the end because I worked odd hours and they closed by the time I was free, so, maybe if they had some hours on a Saturday or something.”“I know I sent in all my data but it would’ve been like helpful if they had like a chart to show like the trends, if there were any trends in my blood sugar readings or my weight or blood pressure, you know.”

## 4. Discussion

Based on both quantitative and qualitative data analysis, this study assessed program enrollment and attrition of a large RPM program implemented in Nebraska from 2014 to 2018. Although the quantitative data illustrated the magnitude of these issues, the qualitative feedback from the patients highlighted patient perceptions of the program and provided further insights into the factors associated with program enrollment and attrition. The current study went beyond several previous evaluation studies of the same program focused on effectiveness based solely on the quantitative data from the patients who completed the 3-month RPM and coaching [17,20,21], and more comprehensively evaluated program impact by also looking into program reach and attrition using a mixed-methods approach [22].

Despite the documented effectiveness of telemedicine in diabetes management [11], non-participation in telemedicine programs remained high among patients with T2D. One recent study of telemedicine utilization among patients with diabetes in California during the period of COVID-19 lockdown revealed that 57% of these patients declined to use telemedicine for diabetes management [23]. This contrasted with the 13% non-participation rate in the RIISCC program where only high-risk patients with T2D recently discharged from the hospital were recruited. Qualitative feedback from these patients identified hectic schedules and fulfilling other duties in life as the most important reason for non-participation. Similar findings were also reported in two large clinical trials based on telemedicine interventions whereby participants cited being busy as one of the top reasons for non-participation [24]. This partially explained our observation that female patients were more likely to decline to participate in the RIISCC program; if female patients were more occupied with family duties such as child education, cleaning, and cooking, they might find it difficult to participate in the telemonitoring program. It is also likely that some female patients might have already been constantly monitoring their health on a regular basis themselves, which reduced their motivation for participation, as mentioned by one of the patients in our study.

Besides non-participation, program attrition can also threaten the reach and impact of telemonitoring programs. The attrition rate in home-based telemonitoring programs for chronic disease management can be as high as 55% [10]. About 16% of patients with T2D enrolled in the RIISCC program withdrew from the intervention without completion. Age at baseline was negatively associated with the odds of attrition. This finding was corroborated by a previous study based on an analysis of data from 100,000 patients [8]. Younger patients might have more commitments in life (e.g., jobs, childcare or education, travel) that could distract them from finishing the telemonitoring program.

The finding that poor baseline health, as indicated by HbA1c and blood pressure, was associated with higher odds of program withdrawal deserves attention. It is plausible that patients with poor baseline health might be more likely to encounter certain barriers to RPM participation such as social determinants of health, patient-provider discordance, or lack of cultural tailoring. These issues disproportionately hamper diabetes management among racial and ethnic minority patients. There was evidence that some of the commonly cited reasons for non or incomplete participation in home telemonitoring for diabetes management among Black and Hispanic patients included disinterest, perceived inconvenience, or lack of perceived benefit of these programs [25]. It may also be that patients with poor baseline health have more severe comorbidities or a more complicated health history, which may lead to program withdrawal due to disease management fatigue or competing priorities in managing multiple chronic conditions. While the nurse coaches in RIISCC had experience in diabetes management, it might be beyond their clinical expertise to advise patients with complex or severe comorbidities. This explains why one patient expressed a preference for talking to a nurse practitioner. Future telemonitoring programs can increase program retention by paying special attention to the needs of patients with high HbA1c or blood pressure at the baseline.

Overall, the RIISCC program was effective, as indicated by substantial, positive changes in HbA1c and patient activation in diabetes management from the baseline to the end of the program and by the positive feedback regarding program staff and remote monitoring equipment from the patients who completed the intervention. The general perception of the program by patients in the Withdrawal group was also positive. Despite these findings, one negative change in health outcome during the RPM program was the increased prevalence of hypertension from the baseline to the end of the intervention. Since patients in the RIISCC were all recently discharged from hospital, the consistent monitoring and control of blood pressure during the hospital stay could have contributed to the relatively lower blood pressure at the baseline. As the RPM program focused mostly on the management of blood glucose, the monitoring and control of blood pressure might not receive similar attention during the program. Findings from our study call for more attention to hypertension management in future RPM programs serving patients with T2D.

One overarching theme cutting across all three groups of patients in this study concerns the technology used in the RIISCC program. According to the Technology Acceptance Model [26], patients’ intention to use a new technology for disease management, including various technologies in RPM, is a function of two factors: perceived usefulness and perceived ease of use. The monitoring equipment used in the RIISCC program were developed more than 10 years ago, and might look cumbersome and not as user-friendly when compared to the current prevailing technology. This was reflected by related feedback from patients from both the Withdrawal and Completed groups, where some concerns over the quality and accuracy of the monitoring equipment were expressed. For patients who were already experienced and knowledgeable about self-monitoring and management of glucose levels, they presumably will not have much motivation to participate in a telemonitoring program unless their perceived unique benefits of participation outweigh perceived costs. This is especially important as many patients in the study singled out being busy in life had made it difficult for them to commit to program participation. With constant advancements in digital technology such as the increasing use and development of wearable, non-invasive blood glucose and blood pressure monitoring [27,28], remote monitoring of diabetes and hypertension will become more efficient, affordable, and user-friendly over time, which will lead to better program reach, retention, and impact.

### Limitations of the Study

Several limitations of this study merit comments. First, the RIISCC program was established as a quality improvement initiative, not a clinical trial, which has limited both the breadth and depth of the data used in this study. For example, there were no comparison groups or data on patient socioeconomic status such as marital status, education, and employment status, which may have impacted our findings. Moreover, the lack of quantitative data on patients who declined to participate from the beginning restricted our capacity in identifying factors contributing to non-participation from a statistical perspective. Second, the modest duration of telephone interviews with patients limits the richness and depth of the qualitative data. Thirdly, part of the variations in patient outcomes during the intervention, including drop-offs, might be related to the performance of the nurse coaches. A more comprehensive program evaluation would benefit from assessing disparities in staff performance and how they might have contributed to related differences in program outcomes. It would help if future program evaluation also incorporated perspectives from nurse coaches and primary care providers to assess program outcomes. Finally, the use of financial incentives in the RIISCC program should have helped with program recruitment and retention, which implies that the participation and retention rates could presumably become worse when no incentives are offered. Caution should be taken before generalizing findings from this study to other situations. Despite these limitations, this study represents a rare effort in adopting a mixed-methods approach to simultaneously evaluate program enrollment, retention, and effectiveness of a large telemonitoring program serving patients with T2D.

## 5. Conclusions

RPM has become more widely adopted and incorporated into diabetes management during and after the COVID-19 pandemic. The impact of an RPM program for diabetes management is not only determined by its effectiveness, but also by its program reach and retention. Being younger or having higher blood glucose or blood pressure at the baseline were associated with higher odds of withdrawing from RPM. The top reason patients cited for non-participation or premature withdrawal from the program was having a hectic lifestyle. Advancements in digital technology have the potential of rendering RPM more efficient, affordable, and user-friendly, which can lead to increased program reach, retention, and impact. Future RPM programs can increase program retention by becoming more responsive to the health needs of vulnerable patients who struggle with managing their diabetes or related comorbidities at the baseline.

## Figures and Tables

**Table 1:** Questions used in interviewing three groups of T2D patients in the RIISCC.

Enrolled Patients Who Completed the Program	Enrolled Patients Who Withdrew from the Program	Eligible Patients Who Declined to Participate in the Program
What motivated you to join this program?	What made you join this program in the beginning?	How did you hear about the program?
What is your overall impression of the program?	What is your overall impression of the program?	Do you think you had all the information you needed before you made your decision to not participate in the program?
Which part or element of the program did you like the most?	Which part or element of the program did you like the most?	Could you share with us why you decided NOT to participate in the program?
How did you like the equipment you used at home during your participation in the program?	How did you like the equipment you used at home when you were in the program?	Looking back, is there anything we could do differently to better motivate your interest in the program?
How do you feel about the services provided by our nurses?	How do you feel about the services provided by our nurses?	If this program was offered to you again in the future, how likely is it would you be willing to participate? Why?
If you could change the program, what would you change?	Why were you unable to complete the program?	Do you have other comments or recommendations for us to consider?
If this program was offered to you again in the future, how likely is it would you be willing to participate? Why?	If you could change the program, what would you change?	
For now the program is offered to eligible patients for free. Would you be willing to participate in the program if you had to pay a certain amount of co-pay or deductible?	If this program was offered to you again in the future, how likely is it would you be willing to participate? Why?	
Do you have other comments or recommendations for the program?	Do you have other comments or recommendations for the program?	

**Table 2:** Patient baseline characteristics by enrollment status in the RIISCC.

Variables	All n = 1993	Completed n = 1420	Withdrawal n = 317	Declined n = 256	*p*-Value
Age, mean (SD)	58.5 (12.5)	59.5 (11.7)	54.5 (13.6)	58.4 (14.6)	<0.001
Female, N (%)	1122 (56.3)	776 (54.6)	173 (54.6)	173 (67.6)	0.001
Race, N (%)					<0.001
Non-Hispanic Whites	1290 (64.7)	961 (67.7)	189 (59.6)	140 (54.7)	
Not white	700 (35.1)	457 (32.2)	127 (40.1)	116 (45.3)	
HbA1c level, mean (SD)	7.8 (2.0)	7.7 (2.0)	8.1 (2.1)	-	0.001
HbA1c > 9, N (%)	372 (18.7)	279 (19.6)	93 (29.3)	-	<0.001
Weight, mean (SD)	225.6 (53.8)	224.7 (52.9)	244.4 (68.6)	-	0.006
High blood pressure, N (%)					0.018
No	1284 (64.4)	1073 (75.6)	210 (66.2)	-	
Yes	435 (21.8)	342 (24.1)	93 (29.3)	-	
PAM score category, N (%)					0.982
Level 1 (≤47)	165 (8.3)	124 (8.7)	28 (8.8)	-	
Level 2 (47.1–55.1)	335 (16.8)	261 (18.4)	58 (18.3)	-	
Level 3 (55.2–67.0)	591 (29.7)	464 (32.7)	99 (31.2)	-	
Level 4 (≥67.1)	671 (33.7)	530 (37.3)	120 (37.9)	-	

**Table 3:** Factors associated with withdrawing from the RIISCC before completion based on results from logistic regression (n = 1663).

Variables	Odds Ratio	95% CI	*p*-Value
Age at baseline	0.97	0.96–0.98	<0.001
Sex			
Female	1.00		
Male	1.03	0.79–1.34	0.819
Race/ethnicity (ref = non-Hispanic white)			
Non-Hispanic White	1.00		
Racial and Ethnic/Minority	1.25	0.95–1.65	0.110
HbA1c (%) at baseline	1.07	1.01–1.14	0.028
Having high blood pressure at baseline			
No	1.00		
Yes	1.39	1.05–1.84	0.022
Baseline patient activation score			
Level 1 (≤47.0)	1.00		
Level 2 (47.1–55.1)	0.94	0.56–1.58	0.815
Level 3 (55.2–67.0)	0.95	0.59–1.53	0.822
Level 4 (≥67.1)	0.99	0.62–1.60	0.979

**Table 4:** Comparison of outcomes at the baseline and the posttest for T2D patients in the RIISCC.

Outcomes	n	Baseline	End of the Intervention	*p*-Value
Weight (pound), mean (SD)	1299	224.7 (52.9)	222.5 (52.4)	<0.001
HbA1c, mean (SD)	1420	7.7 (2.0)	7.1 (1.5)	<0.001
HbA1c > 9%, N (%)	1420	279 (19.6)	151 (10.6)	<0.001
High blood pressure, N (%)	1328	318 (23.9)	432 (32.5)	<0.001
Patient activation score, mean (SD)	1335	64.5 (15.2)	70.12 (15.3)	<0.001

## Data Availability

Due to the need of protecting patient privacy, the data used in this study are not publicized. De-identified data may be requested by contacting the corresponding author at dejun.su@unmc.edu with approval from Nebraska Medicine.

## Abbreviations

The following abbreviations are used in this manuscript:

RPM	remote patient monitoring
T2D	type 2 diabetes mellitus
RIISC	remote interventions improving specialty care
CROC	clinical research outreach coordinator
HbA1c	hemoglobin A1c
PAM-13	The Patient Activation Measure-13
